# Exploring the Hemorrhagic Manifestations of an Adolescent With Dermatosparaxis-Type Ehlers-Danlos Syndrome: A Case Report

**DOI:** 10.7759/cureus.108459

**Published:** 2026-05-07

**Authors:** Akshaya Kumar Balagopal, Santhi T S, Ashokraj Selvam, Vimalraj Vijayakumar

**Affiliations:** 1 General Medicine, Madras Medical College, Chennai, IND; 2 Internal Medicine, Madras Medical College, Chennai, IND; 3 Medical Gastroenterology, Madras Medical College, Chennai, IND

**Keywords:** adamts2, collagen vascular disorder, dermatosparaxis, ehler danlos syndrome, recurrent gi bleed

## Abstract

A 15-year-old female patient presented with recurrent episodes of hematemesis over six months, with no identifiable source on initial evaluations, including upper gastrointestinal (GI) endoscopy and abdominal angiography. Despite a thorough workup for bleeding diatheses, the etiology remained elusive until whole-genome sequencing revealed a homozygous mutation in the ADAMTS2 gene, confirming Ehlers-Danlos syndrome (EDS), dermatosparaxis type (dEDS). Although bleeding is more common in vascular EDS, recurrent GI bleeding in this patient likely resulted from vessel wall weakness and defective collagen processing associated with dEDS. Management included celiprolol, vitamin C, multivitamins, and multidisciplinary follow-up. This case underscores the importance of genetic testing in diagnosing atypical connective tissue disorders and highlights a multidisciplinary approach for managing complex cases with recurrent, unexplained bleeding.

## Introduction

Ehlers-Danlos syndrome (EDS) comprises a heterogeneous group of inherited connective tissue disorders resulting from pathogenic variants in several genes responsible for maintaining extracellular matrix integrity. It is characteristically associated with generalized joint hypermobility, increased skin extensibility, and fragility of connective tissues across multiple organ systems [1]. The 2017 international classification recognizes 13 subtypes of EDS with an estimated prevalence of approximately one in 5,000 individuals [1]. Among these, the hypermobile form is the most frequently encountered, whereas the vascular and dermatosparaxis variants remain quite rare [2]. Dermatosparaxis-type EDS (dEDS) arises from mutations in the ADAMTS2 gene, which impair normal collagen processing. These defects result in marked skin fragility, a tendency toward easy bruising, and occasionally bleeding complications. The condition follows an autosomal recessive pattern of inheritance, contributing to its rarity and making clinical diagnosis challenging [3]. Because of its uncommon occurrence and heterogeneous clinical manifestations, confirmation of the diagnosis often requires genetic testing like next-generation exome sequencing. In this report, we describe a rare case involving a female adolescent diagnosed with dEDS who presented with persistent hematemesis with mucocutaneous bleeding. This case depicts the diagnostic and management challenges associated with rare multisystem genetic disorders.

## Case presentation

History

**Figure 1:**
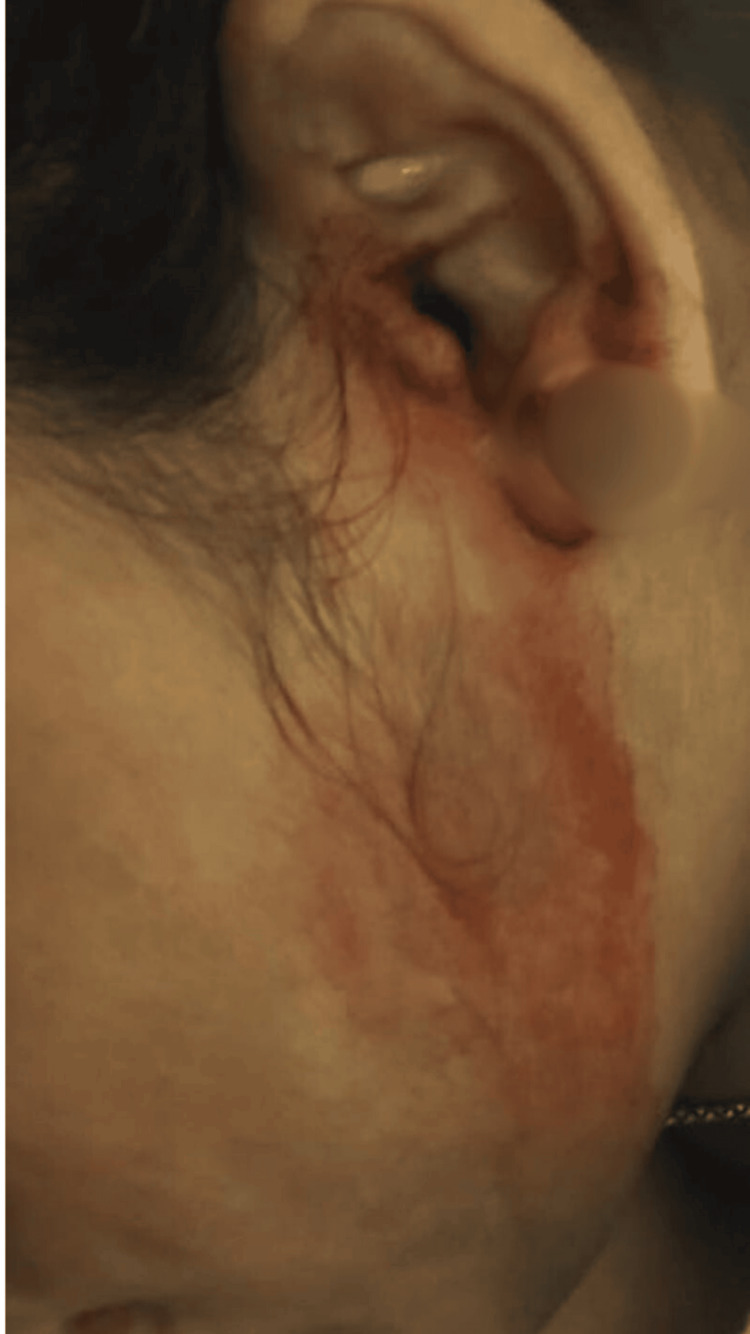
Spontaneous bleeding from the external auditory canal (otorrhagia) without antecedent trauma or local pathology. No otoscopic abnormality was identified

**Figure 2:**
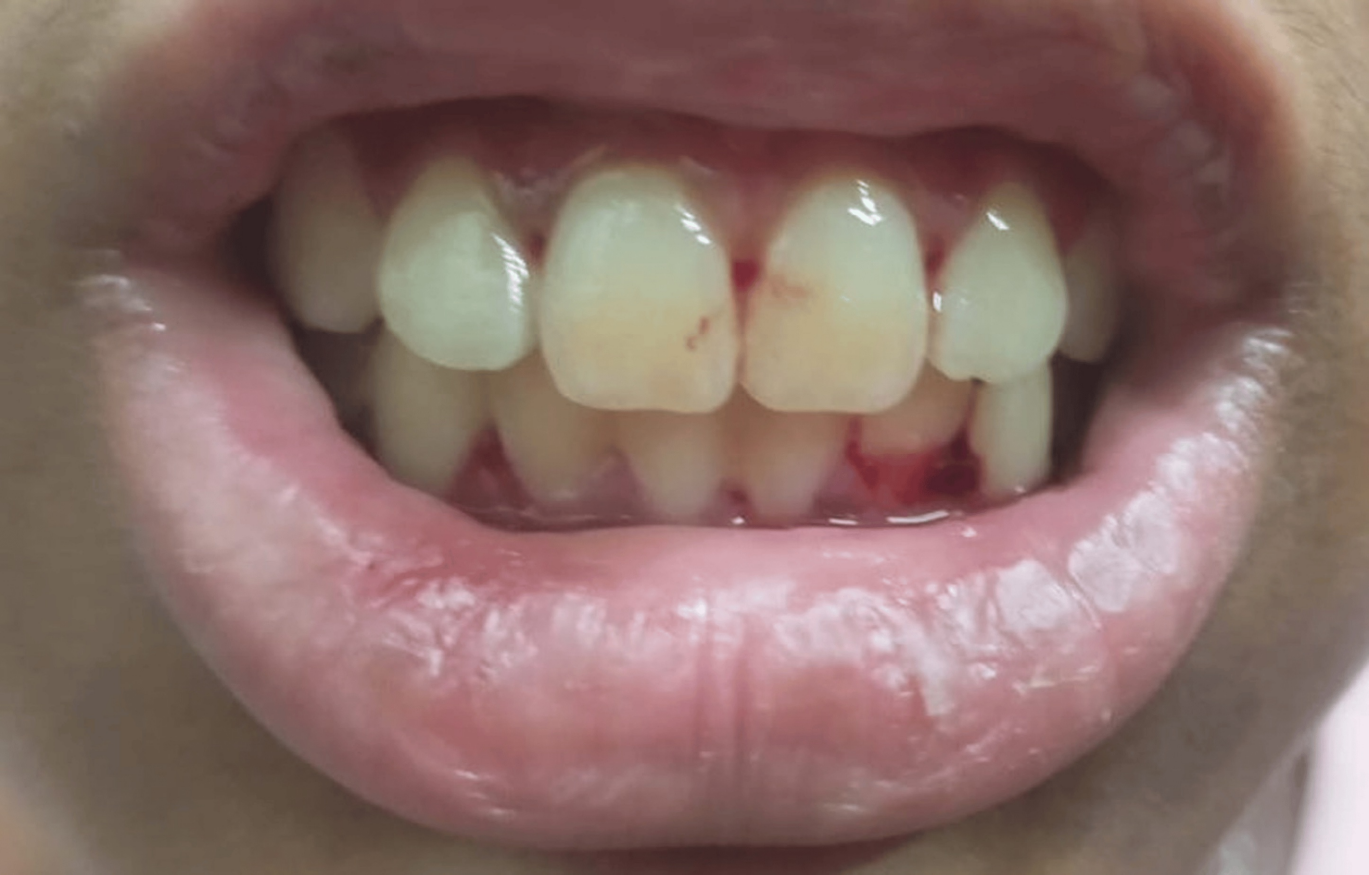
Non-traumatic gingival bleeding demonstrating mucosal fragility characteristic of EDS EDS: Ehlers-Danlos syndrome

**Figure 3:**
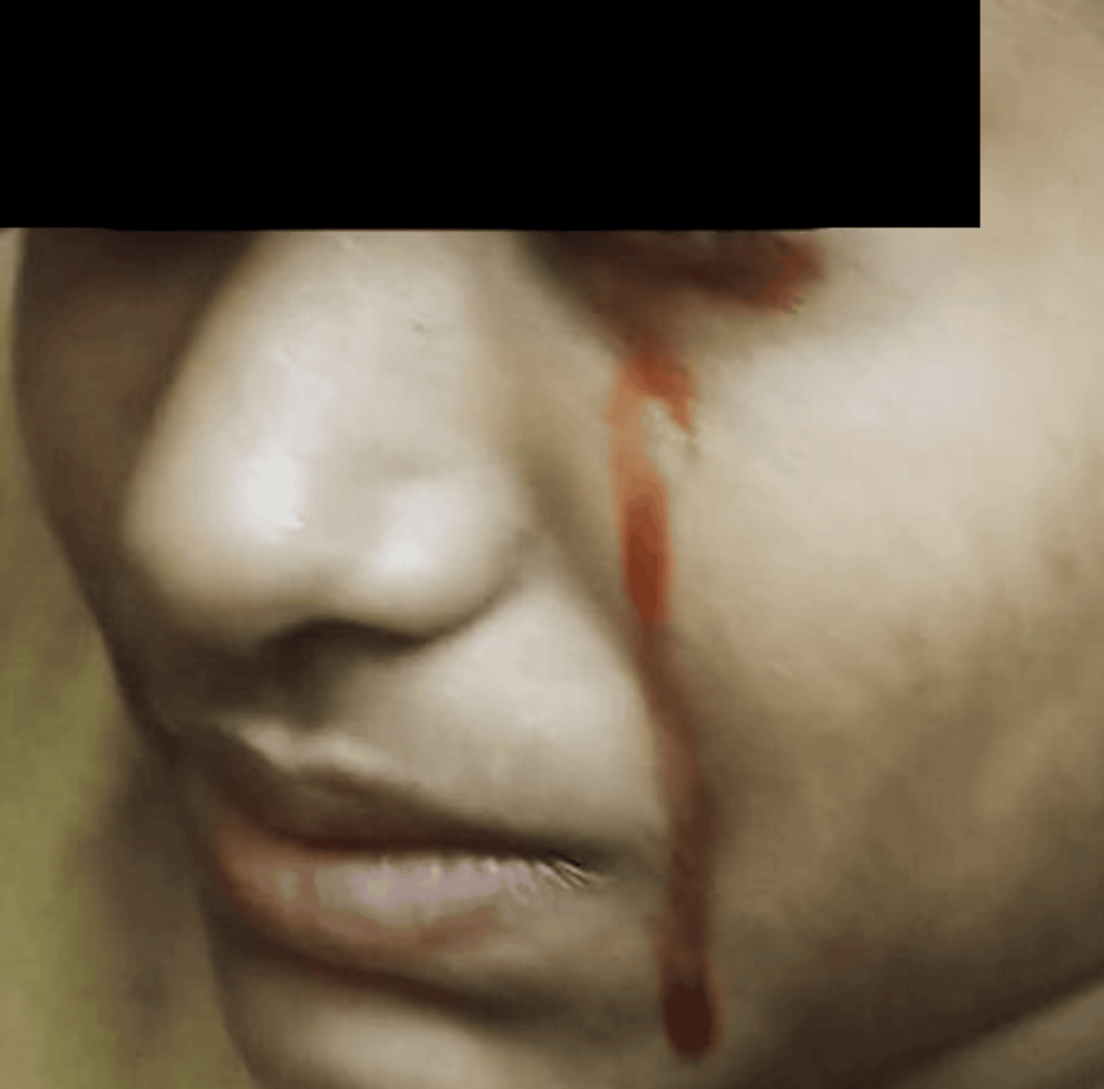
Bloody lacrimation (hemolacria) - an extremely rare hemorrhagic manifestation. Ophthalmic evaluation excluded local causes

A 15-year-old female patient presented with epistaxis and otorrhagia (Figure 1) with a recent history of multiple episodes of hematemesis over six months and hematuria for two months. She had frequent episodes of epistaxis, gingival bleeding (Figure 2), and otorrhagia and a single episode of hemolacria (Figure 3). She also had multiple oral ulcers, polyarthralgia, and increased hair fall. The patient had a history of easy bruisability from minor trauma, with the formation of subcutaneous hematomas in the gluteal region and delayed wound healing. Past surgical history was significant for dislocation of the left thumb at one year of age and developmental dislocation of the hip, for which trochanteric epiphysiodesis was done at seven years of age. Born of a second-degree consanguineous marriage, her father had died of sudden cardiac arrest at around 50 years of age. She had neither comorbidities nor a history of substance abuse.

Examination

The cardiovascular, pulmonary, abdominal, and neurological examinations were within normal limits. The musculoskeletal examination showed normal muscle tone and reflexes. Physical examination revealed the patient had a hypoplastic chin, hypermobility of joints with a Beighton score (tissue hyperextensibility score) of 9, and positive Gorlin’s sign [4]. Dermatological examination showed doughy skin with hyperextensibility, skin translucency, and increased skin fragility with multiple purpura and atrophic scars noted on the thighs. Tenderness was noted on the bilateral shoulder, elbow, and knee joints.

Investigations

Complete blood count, basic metabolic panel, lipid panel, and coagulation profile were normal. Factor assay and rotational thromboelastometry were within normal limits. Immunological evaluation was negative. Ophthalmological evaluation of local causes of hemolacria was negative. Whole-exome sequencing was done, which showed a homozygous missense mutation of exon 17 of the ADAMTS2 gene, a variant of uncertain significance, which was consistent with the clinical diagnosis of dEDS (type VIIC). This aforementioned mutation has so far not been reported in dEDS.

Management

She was started on celiprolol (100 mg daily), vitamin C, and multivitamins, and regular follow-up showed symptomatic improvement with a decrease in frequency of hemorrhage.

## Discussion

dEDS is exceptionally rare, with only 15 cases documented in the literature as of 2026. Among these reports, just one case involving an adolescent was described by Reardon et al. in 1995 [5], while another was identified in a geriatric patient [6]. No other patient has been reported to have presented with the combination of symptoms of hemolacria, otorrhagia, and gingival bleeding, making this a first. The condition results from a deficiency of procollagen N-terminal proteinase, which is essential for normal collagen maturation. dEDS follows an autosomal recessive inheritance, typically arising from homozygous or compound heterozygous mutations in the ADAMTS2 gene located on chromosome 5q35 [5].

Clinical criteria

The diagnosis of dEDS is established using the criteria outlined in the expanded 2017 classification and subsequently confirmed through genetic analysis. The major and minor clinical features associated with dEDS are summarized below (Table 1). According to the proposed diagnostic framework, the minimum criteria require the presence of marked skin fragility (major clinical criterion 1) and characteristic craniofacial features (major clinical criterion 2), along with either one additional major clinical criterion or at least three minor clinical criteria [7].

**Table 1:** Major and minor clinical criteria for the diagnosis of Ehlers-Danlos syndrome, dermatosparaxis type Table taken from Malfait et al. [7] Reproduced with permission from the original authors and Wiley

Major clinical criteria	Minor clinical criteria
1) Extreme skin fragility with congenital or postnatal tears	1) Soft and doughy skin texture
2) Characteristic craniofacial features	2) Skin hyperextensibility
3) Redundant, almost lax skin, with excessive skin folds at the wrists and ankles	3) Atrophic scars
4) Increased palmar wrinkling	4) Generalized joint hypermobility
5) Severe bruisability with risk of subcutaneous hematoma	5) Complications of visceral fragility, such as bladder or diaphragmatic rupture
6) Umbilical hernia	6) Delayed motor development
7) Postnatal growth retardation	7) Osteopenia
8) Short limbs	8) Hirsutism
9) Perinatal complications due to connective tissue fragility	9) Tooth abnormalities
	10) Refractive errors
	11) Strabismus

In the present case, the patient demonstrated extreme skin fragility (major criterion 1), a hypoplastic chin (major criterion 2), pronounced bruising with subcutaneous hematoma formation (major criterion 5), and shortened limbs (major criterion 8). Additional findings included soft, doughy skin (minor criterion 1), increased skin hyperextensibility (minor criterion 2), and generalized joint hypermobility (minor criterion 4). Overall, the patient fulfilled four of the nine major criteria and three minor criteria, thereby meeting the clinical threshold for a diagnosis of dEDS according to the criteria proposed by Malfait et al. [7].

Other system manifestations

Skin fragility in individuals with EDS VIIC can be particularly pronounced, resembling the marked cutaneous fragility described in dermatosparactic cattle [8]. The attachment between the skin and the underlying subcutaneous tissue is often markedly lax, creating a sensation that the skin separates easily when traction is applied.

The increased extensibility and elasticity of connective tissues in EDS contribute to excessive joint mobility and increased distractibility. As a result, affected individuals are predisposed to various orthopedic complications like subluxations, dislocations, and tendon ruptures [9]. Both large and small joints may be involved, and the manifestations often become apparent once the child begins walking. Although skeletal radiographs are typically normal, patients frequently experience chronic joint pain [10].

In addition to vascular fragility resulting from inadequate connective tissue support within vessel walls, the inherent skin fragility contributes to easy bruising, subcutaneous hematoma formation, and other bleeding manifestations in these patients [11]. Greater severity of platelet dysfunction has been associated with a progressively higher bleeding risk, with the majority of cases involving defects in the adenosine diphosphate (ADP)-mediated platelet pathway [12].

The gastrointestinal tract is not spared in EDS, with a spectrum ranging from structural complications - including hiatus hernia, rectocele, visceroptosis, and rectal prolapse - to motility disturbances and, in severe cases, arterial dissections, spontaneous bowel perforation, and rupture of internal organs [13]. In the present case, the patient presented with hematemesis, which raised concerns regarding the safety of performing an upper gastrointestinal endoscopy. Invasive procedures such as colonoscopy should be undertaken only when absolutely necessary and preferably in centers with experienced surgical support available [14].

Treatment

Pharmacologic management in EDS includes agents such as celiprolol, ascorbic acid, zinc, and DDAVP (desmopressin). Celiprolol, as studied in the BBEST (Beta-Blockers in Ehlers-Danlos Syndrome Treatment) trial, functions as a vasodilator with β1-blocking and β2-agonist properties. This combination lowers the heart rate as well as mean and pulse pressures, thereby decreasing stress-related fragility of collagen fibers within the vessel walls [15]. The use of ascorbic acid and zinc remains largely anecdotal. DDAVP has been used on a trial basis in patients with chronic bruising, epistaxis, and perioperative bleeding, given its established role in shortening bleeding time [16].

## Conclusions

This case reinforces the importance of considering collagen vascular disorders and hypermobility syndromes in any patient with unexplained bleeding and a normal hemostatic workup. Reaching the diagnosis here was possible only through careful clinical assessment and an exhaustive evaluation including genetic testing. Alongside diagnosis, structured interdisciplinary care is vital in reducing complications and improving this young patient's quality of life. In summary, this is a valuable case report, with a unique presentation of dEDS, thereby contributing to the limited literature.
